# A Ribosomal S-6 Kinase–Mediated Signal to C/EBP-β Is Critical for the Development of Liver Fibrosis

**DOI:** 10.1371/journal.pone.0001372

**Published:** 2007-12-26

**Authors:** Martina Buck, Mario Chojkier

**Affiliations:** 1 Department of Medicine and Moores Cancer Center, University of California at San Diego, La Jolla, California, United States of America; 2 Department of Medicine, Veterans Affairs (VA) Healthcare Center, San Diego, California, United States of America; Dresden University of Technology, Germany

## Abstract

**Background:**

In response to liver injury, hepatic stellate cell (HSC) activation causes excessive liver fibrosis. Here we show that activation of RSK and phosphorylation of C/EBPβ on Thr217 in activated HSC is critical for the progression of liver fibrosis.

**Methodology/Principal Findings:**

Chronic treatment with the hepatotoxin CCl_4_ induced severe liver fibrosis in C/EBPβ^+/+^ mice but not in mice expressing C/EBPβ-Ala217, a non-phosphorylatable RSK-inhibitory transgene. C/EBPβ-Ala217 was present within the death receptor complex II, with active caspase 8, and induced apoptosis of activated HSC. The C/EBPβ-Ala217 peptides directly stimulated caspase 8 activation in a cell-free system. C/EBPβ^+/+^ mice with CCl_4_-induced severe liver fibrosis, while continuing on CCl_4_, were treated with a cell permeant RSK-inhibitory peptide for 4 or 8 weeks. The peptide inhibited RSK activation, stimulating apoptosis of HSC, preventing progression and inducing regression of liver fibrosis. We found a similar activation of RSK and phosphorylation of human C/EBPβ on Thr266 (human phosphoacceptor) in activated HSC in patients with severe liver fibrosis but not in normal livers, suggesting that this pathway may also be relevant in human liver fibrosis.

**Conclusions/Significance:**

These data indicate that the RSK-C/EBPβ phosphorylation pathway is critical for the development of liver fibrosis and suggest a potential therapeutic target.

## Introduction

The annual worldwide mortality from liver cirrhosis is approximately 800,000 [1], and there is no available treatment [2]. Excessive tissue repair in chronic liver diseases induced by viral, toxic, immunologic, and metabolic disorders [3], results in the deposition of scar tissue and the development of cirrhosis [4]. Quiescent hepatic stellate cells (HSC) produce negligible amounts of extracellular matrix proteins (ECM), but after their activation, these cells develop a myofibroblastic phenotype, proliferate and become the main contributors of ECM [5] [6]. This step is required for the development of liver fibrosis and cirrhosis [7]–[9].

The mitogen-activated protein kinase (MAPK) pathway, through the extracellular signal-regulated kinase (ERK1/2), activates RSK [10]–[14], resulting in the phosphorylation of mouse C/EBPβ (NP_034013 XP_916631) on Thr217 (Thr266 in human C/EBPβ) [5] [10]. The RSK pathway may be critical for HSC activation induced by liver injury, because expression of a catalytically inactive mutant RSK [15], blocked proliferation and survival of cultured HSC upon their activation by collagen type 1 [10]. The RSK phosphoacceptor site in C/EBPβ is identical in mouse and human, it is evolutionarily conserved [5], and essential for survival of activated HSC [10].

Here we show that activation of RSK and phosphorylation of C/EBPβ on Thr217 in activated HSC is critical for the progression of liver fibrosis. Chronic exposure to the hepatotoxin CCl_4_ can induce liver cirrhosis in humans, and it is a classical method of inducing liver injury and fibrosis in mice [10] [16]. We used this model to investigate the role of RSK and phosphorylation of C/EBPβ on Thr217 in liver fibrosis. The hepatotoxin CCl_4_ induced severe liver fibrosis in C/EBPβ^+/+^ mice but not in mice expressing C/EBPβ-Ala217, a non-phosphorylatable RSK-inhibitory transgene. C/EBPβ-Ala217 was present within the death receptor complex II, with active caspase 8, and induced apoptosis of activated HSC. C/EBPβ^+/+^ mice with severe liver fibrosis induced by an 8-week CCl_4 _treatment, while continuing on CCl_4_, were treated with a cell permeant RSK-inhibitory peptide for 4 or 8 weeks. The peptide inhibited RSK activation, stimulating apoptosis of HSC, preventing progression and inducing regression of liver fibrosis compared to control mice treated with CCl_4_. We found similar activation of RSK and phosphorylation of human C/EBPβ (NP_005185) on Thr266 (identical human phosphoacceptor) in activated HSC in the liver of patients with severe liver fibrosis. These data indicate that the RSK-C/EBPβ phosphorylation pathway is critical for the development of liver fibrosis, and that inhibition of the RSK pathway is a potential therapeutic strategy for the prevention and treatment of liver cirrhosis.

## Results

### Mice expressing the RSK-inhibitory C/EBPβ-Ala217 transgene are resistant to hepatotoxin-induced liver fibrosis

Given the important role of RSK in the activation of HSC [10], we hypothesized that a RSK-inhibitory transgenic protein would block phosphorylation of C/EBPβ on Thr217, induce HSC apoptosis and decrease liver fibrosis following chronic liver injury. Because the chronic exposure to the hepatotoxin CCl_4_ can induce liver cirrhosis in humans, and it is a classical method of inducing liver injury and fibrosis in mice [10] [16], we analyzed whether it induces liver cirrhosis in mice expressing the dominant negative, nonphosphorylatable RSK-inhibitory C/EBPβ-Ala217 transgene. This mutation changes the phosphorylatable Thr217 to a nonphosphorylatable Ala217 within the RSK phosphoacceptor site of C/EBPβ. These animals are developmentally normal, fertile and have a normal life span [10], suggesting that the RSK-inhibitory transgene is apparently not toxic.

After the thrice-weekly intraperitoneal administration of CCl_4 _for 12 weeks, we determined the degree of liver fibrosis in coded samples. This is an extensive chronic exposure for mice and comparable to established severe liver fibrosis in humans [17]. We evaluated liver fibrosis employing the following methods: i) microscopic morphology; ii) semi-quantitative METAVIR clinical grading system; iii) collagen type 1 immunofluorescence; iv) quantitative Sirius red collagen-binding assay; v) quantitative hydroxyproline collagen content; vi) RT-PCR for collagen type 1 mRNA; vii) RT-PCR for α-smooth muscle actin (α-SMA) mRNA (present in activated HSC); and viii) RT-PCR for transforming growth factor (TGF-β) mRNA (a pro-fibrotic cytokine) [4] [7] [8].

**Figure 1:**
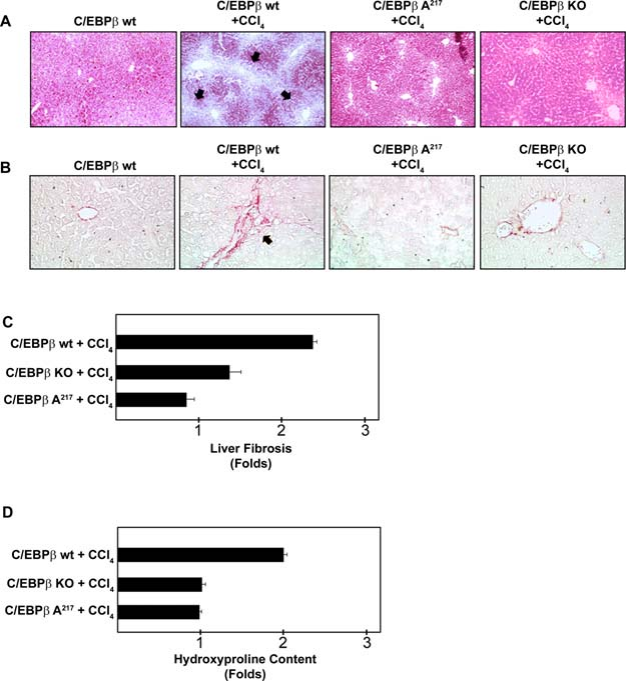
Mice expressing the RSK-inhibitory C/EBPβ-Ala217 transgene are refractory to the induction of liver fibrosis. C/EBPβ^+/+^ [wt], C/EBPβ-Ala217 and C/EBPβ^−/− ^[ko] mice received weekly IP injections of CCl_4_ or mineral oil (control) for 12 weeks as described in Methods. A. Representative Mallory's trichrome stain for liver fibrosis (in blue; arrowheads). All C/EBPβ^+/+^ (wt) mice (*n*: 12) developed severe liver fibrosis. The C/EBPβ-Ala217 (*n*: 12; *P<0.0001*) and C/EBPβ^−/− ^[ko] (*n*: 6; *P<0.01*) mice had either no fibrosis or only minimal liver fibrosis. B. Representative Sirius red immunohistochemistry for collagen (in red; arrowhead). Marked increase in liver collagen in a cirrhotic pattern was observed in C/EBPβ^+/+^, but not in C/EBPβ-Ala 217 or C/EBPβ^−/−^ [ko], mice. C. Analysis of hepatic collagen content by the Sirius red collagen–binding assay, showed a ∼2.5-fold increase in C/EBPβ^+/+^ mice treated with CCl_4_ (*n*: 12), compared to C/EBPβ-Ala217 mice treated with CCl_4_ (*n*: 12; *P<0.001*). C/EBPβ^−/−^ mice were also refractory to the induction of liver fibrosis by CCl_4_ (*n*: 6; *P<0.01*). D. Analysis of hepatic collagen content by the hydroxyproline assay, showed a ∼2-fold increase in C/EBPβ^+/+^ mice treated with CCl_4_ (*n*: 7), compared to C/EBPβ-Ala217 mice treated with CCl_4_ (*n*: 6; *P<0.01*). C/EBPβ^−/−^ mice were also refractory to the induction of liver fibrosis by CCl_4_ (*n*: 6; *P<0.01*).

Liver samples were stained with the classical Mallory's trichrome to identify collagen in the extracellular matrix. The hepatic collagen pattern and content of C/EBPβ^+/+^ (wt) mice treated with CCl_4_ for 12 weeks were similar to those of patients with liver cirrhosis (Figure 1A) [4] [8]. We graded the coded liver samples with the standard 0 to 4 METAVIR clinical system [18], and found that after CCl_4 _treatment, all C/EBPβ^+/+^ mice had severe liver fibrosis (grade 4; *n*: 12), while all C/EBPβ-Ala217 mice had minimal or no liver fibrosis (grade 0; *n*: 6; grade 1; *n*: 6) (*P*<0.0001) (Figure 1A). In agreement with the findings with the Mallory's trichrome, the Sirius red collagen-binding stain also demonstrated decreased liver fibrosis after CCl_4 _treatment in C/EBPβ-Ala217 mice (Figure 1B). Similarly, confocal scanning microscopy with specific antibodies against collagen type 1 also identify decreased liver fibrosis in C/EBPβ-Ala217 mice, compared to C/EBPβ^+/+^ mice, after CCl_4 _treatment (Figure S1). Although the intra- and inter-observer variability in the semi-quantitative analysis of liver fibrosis was low, we confirmed these findings using quantitative analysis of liver fibrosis.

The quantitative analysis of liver collagen, the major extracellular matrix protein in liver fibrosis [4], with the Sirius red collagen-binding assay [19] (Figure 1C), demonstrated that C/EBPβ-Ala217 mice were refractory to the development of liver fibrosis after chronic exposure to the hepatotoxin. The liver collagen content increased approximately 2.5-fold from baseline in C/EBPβ^+/+^ (wt) mice (*P*<0.001) while remaining unchanged in C/EBPβ-Ala217 mice (NS) (Figure 1C). Similarly, chronic administration of CCl_4_ increased liver hydroxyproline collagen content in C/EBPβ^+/+^ (wt) mice (*P*<0.01) while remaining unchanged in C/EBPβ-Ala217 mice (NS) (Figure 1D).

We have reported that by lacking the essential C/EBPβ-PhosphoThr217, C/EBPβ−/− HSC are also unable to survive the activation signals [10]. Thus, we postulated that C/EBPβ−/− mice would be refractory to the induction of liver fibrosis. Indeed, C/EBPβ^−/− ^(ko) mice had a lower fibrotic response than C/EBPβ^+/+^ mice to the chronic treatment with CCl_4_ (*P*<0.01) (Figure 1 A, B, C and D).

**Figure 2:**
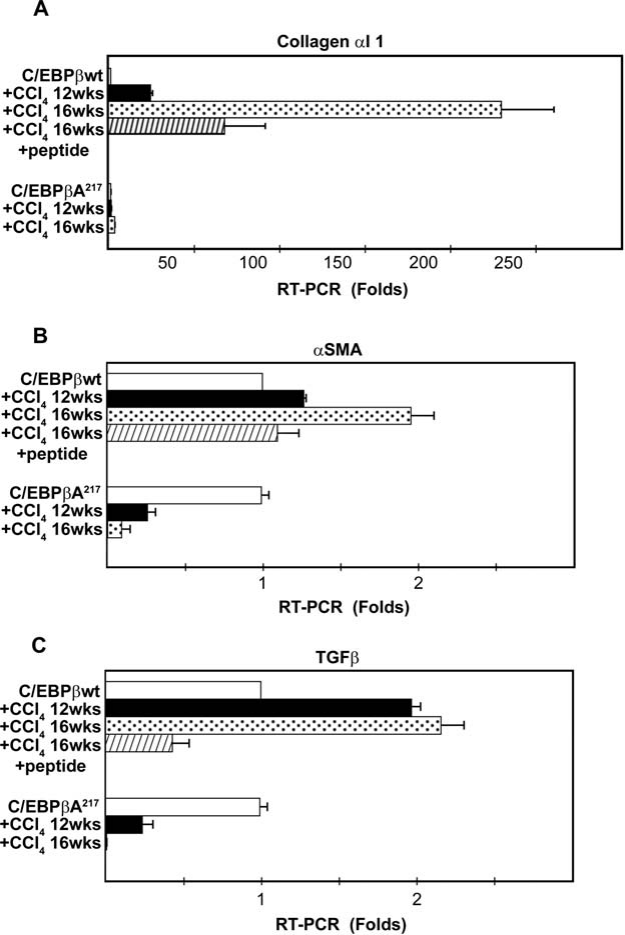
Mice expressing the RSK-inhibitory C/EBPβ-Ala217 transgene are resistant to hepatotoxin-induced liver fibrogenesis. Mice were treated with CCl_4_ or control mineral oil for 12 or 16 weeks and RT-PCR was performed as described in Materials and Methods. A. RT-PCR for collagen α1 type 1 was induced in C/EBPβ ^+/+^ [wt] mice treated with CCl_4_ for 12 or 16 weeks (*P<0.01*). Treatment of these animals after week 8 with the RSK-inhibitory peptide while continuing the exposure to the hepatotoxin, as described in Materials and Methods blocked this increase at week 16 (*P<0.05*). Collagen α1 type 1 was not increased in livers of C/EBPβ-Ala217 mice at 12 or 16 weeks (*P<0.01*). B. RT-PCR for α-SMA was induced in C/EBPβ ^+/+ ^[wt] mice treated with CCl_4 _for 12 or 16 weeks (*P<0.05*). Treatment of these animals after week 8 with the RSK-inhibitory peptide while continuing the exposure to the hepatotoxin, as described in Materials and Methods blocked this increase at week 16 (*P<0.05*). α-SMA was not increased in livers of C/EBPβ-Ala217 mice at 12 or 16 weeks (*P<0.05*). C. RT-PCR for TGF-β was induced in C/EBPβ ^+/+ ^[wt] mice treated with CCl_4 _for 12 or 16 weeks (*P<0.01*). Treatment of these animals after week 8 with the RSK-inhibitory peptide while continuing the exposure to the hepatotoxin, as described in Materials and Methods, blocked this increase at week 16 (*P<0.01*). TGF-β was not increased in livers of C/EBPβ-Ala217 mice at 12 or 16 weeks (*P<0.01*).

Further, the expression of the liver fibrogenic indicators such as collagen α 1 type 1 mRNA (newly synthesized collagen) (NM_007742 )(*P<0.01*), α-SMA mRNA (activated HSC) (NM_007392.2) (*P<0.05*), and TGF-β mRNA (fibrogenic cytokine) (NM_009370.2) (*P<0.01*), were induced by CCl_4 _treatment significantly more in C/EBPβ^+/+^ mice than in C/EBPβ-Ala217 mice as measured by RT-PCR (Figure 2).

In summary, blocking phosphorylation of C/EBPβ-Thr217 through the inhibition of RSK activity with the C/EBPβ-Ala217 transgene or by C/EBPβ gene knock-out decreases the fibrotic response of the liver to chronic injury.

### Mice expressing the RSK-inhibitory C/EBPβ-Ala217 transgene are resistant to hepatotoxin-induced liver inflammation

Because liver inflammation, at least in part through the activation of macrophages [20]–[22] and loss of hepato-trophic factors from HSC and liver endothelial cells [23] [24] is a major contributor to liver injury and liver fibrosis, we assessed the degree of liver injury and inflammation in response to the hepatotoxin. Liver injury was determined by measuring serum alanine aminotransferase (ALT) levels in mice after exposure to the hepatotoxin. Serum ALT is a sensitive and specific indicator of hepatocellular injury in humans and animals, and it is the standard clinical test used by the U.S. Food and Drug Administration to ascertain hepatotoxicity of herbal products and drugs [25] [26]. We found that C/EBPβ-Ala217 mice had less liver injury than C/EBPβ^+/+^ mice after CCl_4_ treatment, judging by the ALT serum levels (Table S1).

Using a microarray assay to assess expression of 66 inflammation genes in the liver of control and transgenic mice, we found that the expression of 21 inflammation genes was decreased, while the expression of other 45 inflammation genes was unchanged in C/EBPβ-Ala217 mice after CCl_4_–induced liver injury, when compared to C/EBPβ^+/+^ animals treated with CCl_4_ (Table 1).

**Table 1:** Decreased Inflammation in the Livers of C/EBPβ- Ala217 mice after CCl_4_ treatment.

#	GeneBank	Symbol	Description	Gene Name	Liver Expression in C/EBPβ- A 217 (% of control)
1	NM_009744	Bcl6	B-cell leukemia/lymphoma 6	Bcl5	16
2	NM_009807	Casp1	Caspase 1	ICE/IL 1bc	19
3	NM_011331	Ccl12	Chemokine (C-C motif) ligand 12	MCP-5/Scya12	32
4	NM_011888	Ccl19	Chemokine (C-C motif) ligand 19	CKb11/ELC	9
5	NM_021443	Ccl8	Chemokine (C-C motif) ligand 8	1810063B20Rik/AB023418	9
6	NM_011338	Ccl9	Chemokine (C-C motif) ligand 9	CCF18/MRP-2	27
7	NM_009915	Ccr2	Chemokine (C-C motif) receptor 2	CC-CKR-2/CCR2A	16
8	NM_009916	Ccr4	Chemokine (C-C motif) receptor 4	Cmkbr4/LESTR	23
9	NM_009142	Cx3cl1	Chemokine (C-X3-C motif) ligand 1	AB030188/ABCD-3	30
10	NM_021704	Cxcl12	Chemokine (C-X-C motif) ligand 12	AI174028/PBSF	11
11	NM_009141	Cxcl5	Chemokine (C-X-C motif) ligand 5	AMCF-II/ENA-78	23
12	NM_008599	Cxcl9	Chemokine (C-X-C motif) ligand 9	BB139920/CMK	9
13	NM_010548	IL10	Interleukin 10	CSIF/IL-10	23
14	NM_008349	IL10rb	Interleukin 10 receptor, beta	6620401D04Rik/AI528744	29
15	NM_010551	IL16	Interleukin 16	mKIAA4048	30
16	NM_008360	IL18	Interleukin 18	Igif/IL-18	22
17	NM_010555	Il1r2	Interleukin 1 receptor, type II	CD121b/IL1r-2	27
18	NM_008368	IL2rb	Interleukin 2 receptor, beta chain	CD122/IL-15Rbeta	32
19	NM_013563	IL2rg	Interleukin 2 receptor, gamma chain	CD132/[g]c	32
20	NM_009263	Spp1	Secreted phosphoprotein 1	AA960535/AI790405	7
21	NM_011798	Xcr1	Chemokine (C motif) receptor 1	Ccxcr1/GPR5	17

Animals received either mineral oil [MO] or CCl_4_. Thirty hours after a single intraperitoneal dose of CCl_4_,

C/EBPβ Ala217 (*n*: 9) mice had a markedly decreased liver expression of inflammatory genes compared to C/EBPβ^+/+^ mice (*n*: 9). A panel of 86 inflammatory genes were evaluated by RT-QPCR; 21 of these genes were significantly reduced (P<0.05 for all 21 genes).

**Figure 3:**
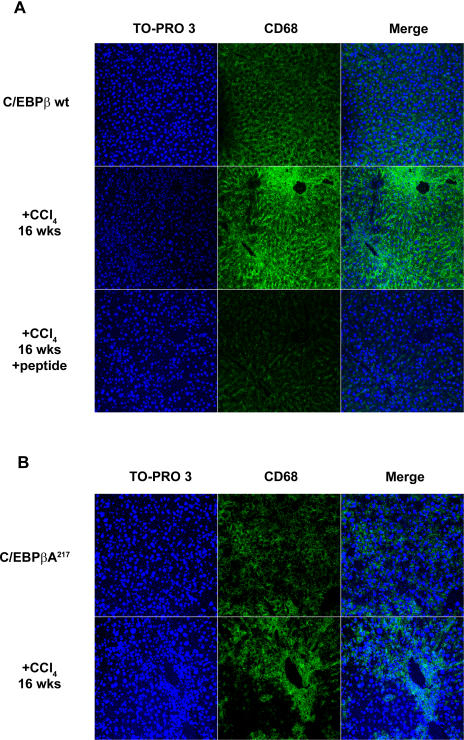
Mice expressing the RSK-inhibitory C/EBPβ-Ala217 transgene are resistant to hepatotoxin-induced liver inflammation. Mice were treated with CCl_4_ or control mineral oil for 16 weeks as described in Materials and Methods. A. Activated monocytes/macrophages, identified by confocal microscopy for CD-68 (green), were increased in livers of C/EBPβ ^+/+^ [wt] mice treated with CCl_4_ for 16 weeks (middle panels). Treatment of these animals after week 8 with the RSK-inhibitory peptide while continuing the exposure to the hepatotoxin, as described in Material and methods, blocked the monocytes/macrophage inflammatory reaction at week 16 (lower panels). Nuclei are identified with TO-PRO-3 (blue). Only background staining was observed when omitting the first antibody. Microscopy shown is representative of six animals in each group. B. Activated monocytes/macrophages, were not increased in livers of C/EBPβ-Ala217 mice as much as in the livers of C/EBPβ ^+/+^ [wt] mice after treatment with CCl_4_ for 16 weeks (lower panel). Microscopy shown is representative of six animals in each group.

These data suggest that partial resistance to liver injury and inflammation may contribute to the prevention of liver fibrosis in C/EBPβ-Ala217 mice. A decreased inflammatory response, mediated at least in part by monocytes/macrophages in the livers of C/EBPβ^+/+^ mice (Figure 3A), may be responsible for the decreased liver injury in C/EBPβ-Ala217 mice, since RSK inhibition also affected the recruitment of CD-68+ inflammatory cells to the liver (Figure 3B).

Given that CCl_4_ is metabolized by the cythocrome-P-450 2E-1(Cyp-2E-1) (ABH07947) to form a free radical hepatotoxic metabolite [27], we evaluated the possibility that C/EBPβ-Ala217 affects Cyp-2E1 expression and/or activity. The Cyp-2E1 mRNA expression was similarly inhibited by CCl_4_ in the livers of C/EBPβ^+/+^ and C/EBPβ-Ala217 mice (Figure S2A). In addition, the Cyp-2E-1 protein expression was also similar in the livers of C/EBPβ^+/+^ and C/EBPβ-Ala217 mice after the hepatotoxin treatment (Figure S2B). Moreover, the Cyp-2E1 activity as measured in a cell-free system was unaltered by a RSK-inhibitory C/EBPβ-Ala217 peptide, even at µM concentrations (Figure S2C).

These results indicate that the protective effects of the RSK-inhibitory transgene in CCl_4_ -induced liver injury and fibrosis are not due to the spurious blockade of the production of a toxic CCl_4_ metabolite.

### Mice expressing the RSK-inhibitory C/EBPβ-Ala217 transgene are resistant to hepatotoxin-induced HSC activation and proliferation

Quiescent HSC produce negligible amounts of ECM, but after their activation, these cells develop a myofibroblastic phenotype, proliferate, and become the main contributors of ECM [5] [6]. Because this step is required for the development of liver fibrosis and cirrhosis [7]–[9], we analyzed the activation and proliferation of HSC in the livers of mice chronically exposed to the hepatotoxin.

**Figure 4:**
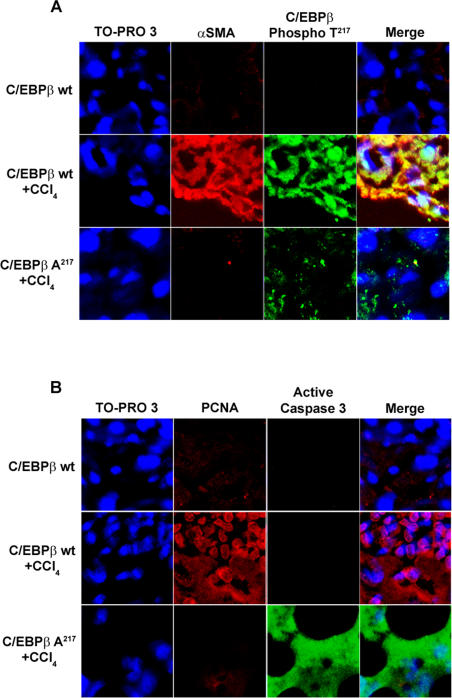
Mice expressing the RSK-inhibitory C/EBPβ-Ala217 transgene are refractory to hepatic stellate cell activation and proliferation. Mice received CCl_4 _or mineral oil injections for 12 weeks as described in Materials and methods. A. Activated stellate cells, identified by confocal microscopy for α-smooth muscle actin (α-SMA; red), displayed C/EBPβ-PhosphoThr217 (green) in livers of C/EBPβ ^+/+^ [wt], but not C/EBPβ-Ala217, mice treated with CCl_4_. Colocalization of α-SMA and C/EBPβ-PhosphoThr217 is shown in yellow (merge). Nuclei are identified with TO-PRO-3 (blue). Only background staining was observed when omitting the first antibody. B. Proliferating cell nuclear antigen (PCNA; red) was present in activated stellate cells only in livers of C/EBPβ^+/+^ [wt] mice treated with CCl_4_, while active caspase 3 (green) was found in HSC only in livers of C/EBPβ-Ala217 mice treated with CCl_4_. Nuclei are identified with TO-PRO-3 (blue).

As expected, chronic CCl_4_ administration to C/EBPβ^+/+^ (wt) mice, induced marked activation of HSC, as indicated by the positive immunofluorescence for α-SMA within the scar tissue [5] [6] (Figure 4A), and proliferation of HSC, as indicated by the presence of proliferating cell nuclear antigen (PCNA; DNA polymerase δ auxiliary protein), an S-phase marker [28] (Figure 4B). By contrast, C/EBPβ-Ala217 mice were refractory to the induction of HSC activation and proliferation by CCl_4_ treatment (Figure 4A and B). Moreover, chronic CCl_4_ treatment induced the apoptotic cascade in HSC in the livers of C/EBPβ-Ala217 mice, but not C/EBPβ^+/+^ mice, as determined by the presence of active caspase 3 immunofluorescence (Figure 4B). After chronic CCl_4_ administration, C/EBPβ was phosphorylated on Thr217 in HSC of C/EBPβ^+/+^ mice, but not in C/EBPβ-Ala217 mice, as determined by confocal microscopy (Figure 4A and B), using specific antibodies against this phosphorylated epitope [10].

**Figure 5:**
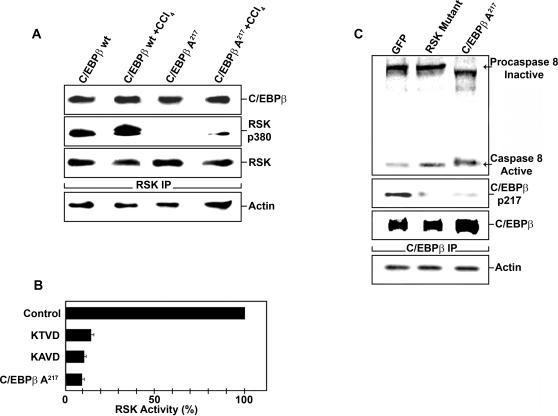
C/EBPβ-Ala217 inhibits RSK activation in hepatic stellate cells. A. A phospho-RSK immunoblot was performed on RSK immunoprecipitates from protein lysates of purified HSC in an experiment conducted as described in (Fig. 1). Phosphorylated RSK (RSKp380) was decreased in HSC isolated from C/EBPβ-Ala217 mice. C/EBPβ and RSK were similar in the different experimental groups. β-Actin was used as an internal control for the immunoprecipitations. Results from triplicate samples of three independent experiments are shown. B. RSK activity was determined in a cell-free system as described in Methods. Recombinant RSK was activated with ATP (125 µM) in the presence or absence of Ac-KThrVD-CHO (200 µM), Ac-KAlaVD-CHO (200 µM), or C/EBPβ216-253-Ala217 (0.25 nM) peptides. Staurosporine was used as a control inhibitor (0.01 nM). All C/EBPβ peptides inhibited RSK activity to a similar extent as staurosporine (P<0.01). Results from triplicate samples of two independent experiments are shown. C. Primary human C/EBPβ^+/+^ HSC were transfected with vectors (1 µg each) expressing green fluorescent protein with control wt RSK (GFP), a dominant negative RSK mutant, or C/EBPβ-Ala217. Transfected HSC were selected by sorting for GFP, and cell lysates were immunoprecipitated with C/EBPβ specific antibodies. C/EBPβ-PhosphoThr217 (C/EBPβp217), and caspase 8 immunoblots were performed in C/EBPβ immunoprecipitates. Dominant negative RSK or C/EPBβ-Ala217 prevented C/EPBβ phosphorylation and stimulated the association of unphosphorylated C/EBPβ with active caspase 8. β-Actin was used as an internal control for the immunoprecipitations. Results from triplicate samples of three independent experiments are shown.

Because the mixed cell population of the liver limits the evaluation of signaling cascades in a specific cell type, we studied the fibrogenic pathway in purified HSC. After chronic CCl_4_ or control mineral oil administration, we analyzed proteins associated with C/EBPβ, immunopurified from HSC, freshly isolated from C/EBPβ^+/+^ and C/EBPβ-Ala217 mice. C/EBPβ-Ala217 binding to, and blocking, RSK phosphorylation (Figure 5A) results in decreased phosphorylation of C/EBPβ on Thr217, and presumably, other target survival proteins by activated RSK [5] [10] [11]. The Ac-KAla217VD-CHO or C/EBPβ216-253-Ala217 (0.25 nM) peptides inhibited RSK activity in a cell-free system, suggesting a direct effect (Figure 5B). The Ac-KThr217VD-CHO wt peptide also inhibited RSK, probably, because it binds to the kinase but cannot be phosphorylated by RSK given its small size (Figure 5B).

### C/EBPβ-Ala217 transgene and unphosphorylated C/EBPβ-Thr217 are associated with active caspase 8 and death receptor complex II proteins in HSC

**Figure 6:**
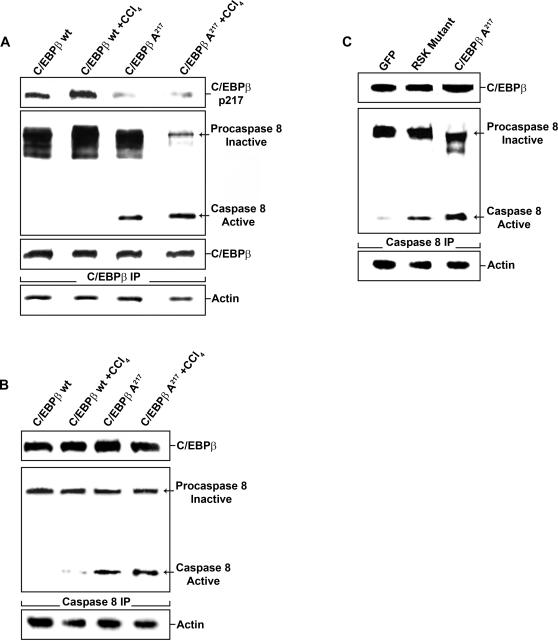
C/EBPβ-Ala217 associates with active caspase 8 in hepatic stellate cells. A. A caspase 8 immunoblot was performed on C/EBPβ immunoprecipitates from protein lysates from samples described in (Fig 5 A). The association between C/EBPβ-Ala217 with active caspase 8 was increased in HSC isolated from C/EBPβ-Ala217 mice. Phosphorylated C/EBPβ-Thr217 was decreased in HSC from C/EBPβ-Ala217 mice treated with CCl_4_. C/EBPβ and RSK were similar in the different experimental groups. β-Actin was used as an internal control for the immunoprecipitations. B. Reciprocal caspase 8 immunoprecipitation of experiment described in (A) confirmed the association of C/EBPβ-Ala217 with active caspase 8. β-Actin was used as an internal control for the immunoprecipitations. C. Reciprocal caspase 8 immunoprecipitation of experiment described in (Figure 5C), confirmed the association of C/EBPβ-Ala217 with active caspase 8. β-Actin was used as an internal control for the immunoprecipitations.

Unexpectedly, we found that C/EBPβ-Ala217 was present with active caspase 8 in HSC from C/EBPβ-Ala217 mice after chronic CCl_4_ administration and, to a lesser extent, after mineral oil administration (Figure 6A). In contrast, the association between inactive procaspase 8 and C/EBPβ-PhosphoThr217 (Figure 6A), as well as that between C/EBPβ and activated phospho-RSK (Figure 5A), increased in HSC of C/EBPβ^+/+^ mice after chronic CCl_4_ administration. Reciprocal immunoprecipitation with caspase 8 antibodies confirmed the presence of C/EBPβ- Ala217 with active caspase 8 (Figure 6A). RSK phosphorylation was inhibited in HSC from C/EBPβ-Ala217 mice, indicating that C/EBPβ-Ala217 not only associates with RSK but that it also decreases its phosphorylation and activation (Figure 5A). The hypothesis that inhibition of RSK by nonphosphorylatable C/EBPβ-Ala217 is critical for caspase 8 activation is supported by the increased caspase 8 activation in HSC from C/EBPβ-Ala217 mice after chronic CCl_4_ administration (Figure 6A).

Next, we studied whether blocking RSK activity results in activation of caspase 8. Activated primary human C/EBPβ^+/+^ HSC were transfected with vectors expressing green fluorescent protein with a control wt RSK (GFP), to facilitate cell sorting, with a dominant negative RSK mutant, or with the RSK-inhibitory dominant negative C/EBPβ-Ala217. The HSC were activated on a collagen type 1 matrix, a condition that recapitulates the activation of HSC in vivo [9], and identified by their expression of GFAP [10].

As expected, C/EBPβ-PhosphoThr266 (identical to mouse phosphoacceptor Thr217) was markedly decreased in activated human HSC expressing either the RSK mutant or C/EBPβ-Ala217. Moreover, we found that unphosphorylated C/EBPβ was associated active caspase 8 (Figure 5C). The RSK phosphoacceptor site in C/EBPβ is identical in mouse and human, it is evolutionarily conserved [5], and essential for HSC survival upon their activation [10].

**Figure 7:**
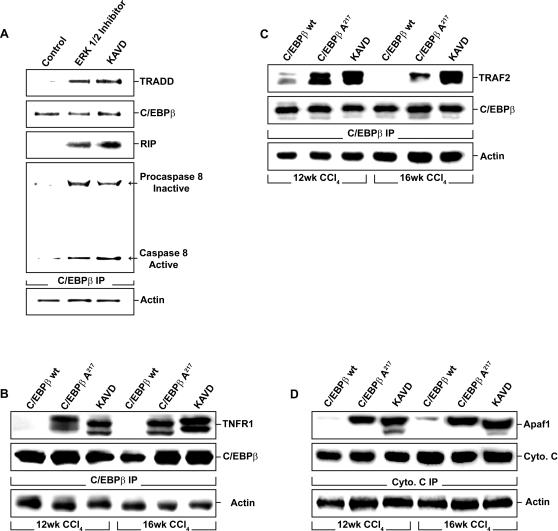
RSK inhibition induces the association of C/EBPβ with active caspase 8, TNFR1, TRAF2, TRADD and RIP. A. TRADD, C/EBPβ, RIP and caspase 8 immunoblots were performed on C/EBPβ immunoprecipitates from primary human HSC treated with an ERK1/2 inhibitor (10 µM) or the cell permeant Ac-KA217VD-CHO peptide (200 µM). Blocking the phosphorylation of C/EBPβ by RSK with the ERK1/2 inhibitor or the cell permeant Ac-KA217VD-CHO (KAVD) peptide, increased the association between C/EBPβ, active caspase 8, TRADD and RIP. β-Actin was used as an internal control for the immunoprecipitations. B. TNFR1 and C/EBPβ immunoblots were performed on C/EBPβ immunoprecipitates from HSC isolated from mice treated with CCl_4_ for 12 or 16 weeks as described in Materials and Methods. Blocking the phosphorylation of C/EBPβ by RSK with C/EBPβ-Ala217 transgene or the cell permeant Ac-KA217VD-CHO peptide increased the association between C/EBPβ and TNFR1. β-Actin was used as an internal control for the immunoprecipitations. C. TRAF2 and C/EBPβ immunoblots were performed on C/EBPβ immunoprecipitates from HSC isolated from mice treated with CCl_4_ for 12 or 16 weeks as described in Materials and Methods. Blocking the phosphorylation of C/EBPβ by RSK with C/EBPβ-Ala217 transgene or the cell permeant Ac-KA217VD-CHO peptide increased the association between C/EBPβ and TRAF2. β-Actin was used as an internal control for the immunoprecipitations. D. Cytochrome C and Apaf1 immunoblots were performed on cytochrome C immunoprecipitates in livers from mice treated with CCl_4_ for 12 or 16 weeks as described in Materials and Methods. Blocking the phosphorylation of C/EBPβ by RSK with C/EBPβ-Ala217 transgene or the cell permeant Ac-KA217VD-CHO peptide increased the association between cytochrome C and Apaf1. β-Actin was used as an internal control for the immunoprecipitations.

C/EBPβ-Ala266 associated with active caspase 8, in cells expressing the dominant negative RSK (Figure 5C), which prevents C/EBPβ phosphorylation on Thr266 by RSK. In contrast, in the control cells expressing GFP and RSK wt, C/EBPβ was phosphorylated and associated with procaspase 8 (Figure 5C), as we reported previously [10]. Reciprocal immunoprecipitation with caspase 8 specific antibodies confirmed the presence of unphosphorylated C/EBPβ with active caspase 8 (Figure 6 B and C). After blocking the RSK-C/EBPβ phosphorylation cascade in activated HSC, with either an ERK1/2 inhibitor or a cell permeant Ac-KAla217VD-CHO peptide, which contains the mutated C/EBPβ-Ala217 phosphoacceptor, unphosphorylated C/EBPβ became associated with other members of the death receptor complex II, such as TNFR1, TRAF2, TRADD and RIP [29] (Figure 7A, B, and C). These associations were identified in C/EBPβ, TNFR1, TRAF2, TRADD and RIP immunoprecipitations (Figure 7 and Figure S3). The Ac-KAla217VD-CHO peptide is cell permeant due to its N-terminus acetyl group, as it has been documented for other peptides used as substrate or inhibitors of caspases [30] [31].

### A cell-permeant C/EBPβ-Ala217 peptide stimulates active caspase 8 and cell death in collagen type 1-induced activation of cultured HSC

We corroborated the activation of caspase 8 in HSC, under conditions that prevented phosphorylation of C/EBPβ, as described above, by demonstrating the association between cytochrome C with Apaf-1 (Figure 7D), which reflects a downstream amplification effect of active caspase 8 on mitochondrial apoptotic pathways [32]. The reciprocal analysis could not be performed since Apaf-1 antibodies are not suitable for immunoprecipitation. Moreover, inhibition of the RSK-C/EBPβ phosphorylation cascade with the cell permeant C/EBPβ-Ala217 peptide, Ac-KAla217VD-CHO induced expression of the apoptosis effector caspase 3 in cultured, activated mouse HSC, but not in primary mouse hepatocytes (Figure S4). These findings suggest a selective induction of apoptotic pathways by C/EBPβ-Ala217 peptides in cultured activated HSC but not in cultured primary hepatocytes. This conclusion is supported by the apoptotic changes in activated HSC but not hepatocytes of C/EBPβ-Ala217 transgenic mice treated with CCl_4_, and by the lack of apoptosis of normal quiescent HSC and hepatocytes of C/EBPβ-Ala217 transgenic mice not treated with CCl_4_. In addition, after CCl_4 _treatment, hepatocyte injury and hepatic inflammation are decreased in C/EBPβ-Ala217 transgenic mice compared to control C/EBPβ^+/+^ mice, arguing for a protective rather than an apoptotic effect on hepatocytes.

**Figure 8:**
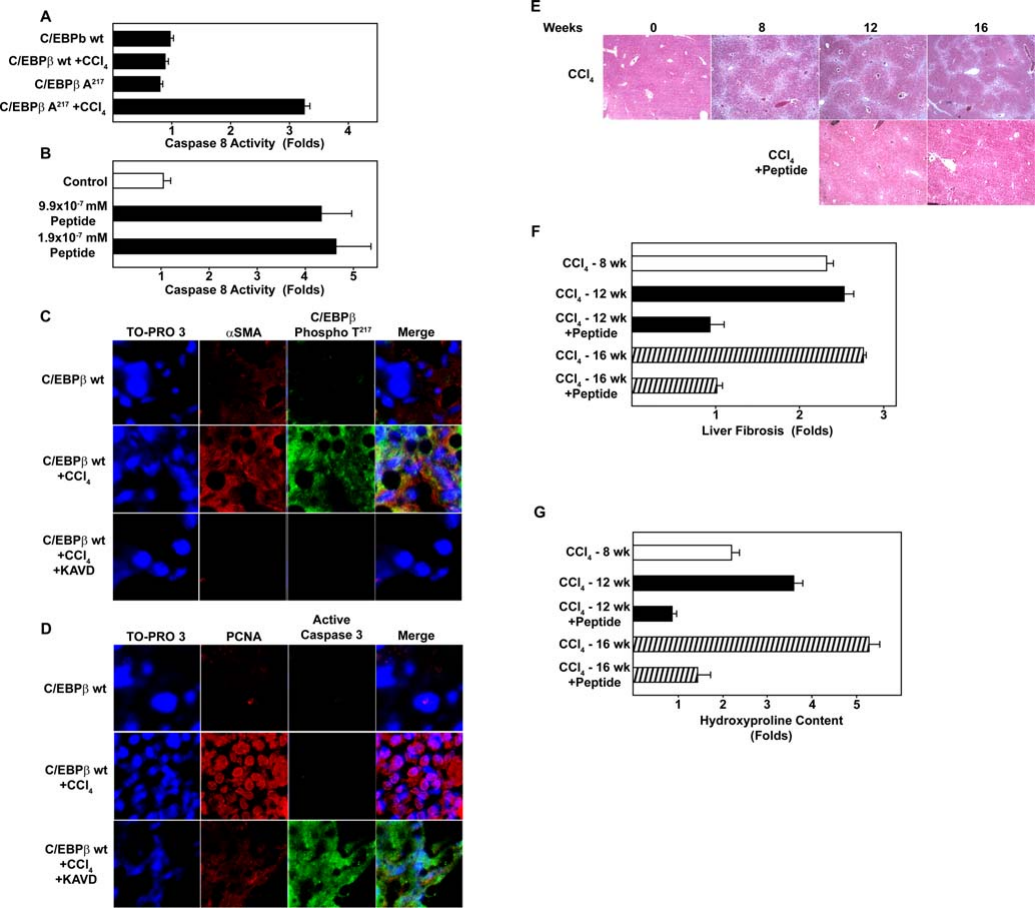
The RSK-inhibitory peptide blocks hepatic stellate cell activation and liver fibrosis induced by CCl_4_. A Caspase 8 activity was measured in lysates from HSC isolated from C/EBPβ^+/+ ^[wt] and C/EBPβ-Ala217 mice untreated or treated with CCl_4_ for 24 hr. Caspase activity was increased in C/EBPβ-Ala217 mice treated with CCl_4_. Results from triplicate samples of two independent experiments are shown. B. Caspase activation in a cell-free system was determined as described in Methods. The Ac-KAla217VD-CHO peptide enhanced the activation of caspase 8 at picomolar concentrations. Baseline caspase 8 activity was 3.8 U (100%). Results from triplicate samples of three independent experiments are shown (*P<0.01* for the Ac-KAla217VD-CHO peptide). C. Animals received a single injection of CCl_4 _or mineral oil as described in Methods. α-SMA (red) and C/EBPβ-PhosphoThr217 (green) were identified as described in Materials and methods. Treatment with the cell permeant Ac-KAla217VD-CHO peptide blocked the expression of α-SMA and C/EBPβ-PhosphoThr217. D. PCNA (red) and active caspase 3 (green) were identified as described in Materials and methods. Treatment with the Ac-KAla217VD–CHO peptide blocked the expression of PCNA and induced active caspase 3. E. C/EBPβ^+/+^ (wt) mice with severe liver fibrosis after treatment with CCl_4_ for 8 weeks, while continuing on CCl_4_, received the RSK inhibitory peptide (5 µg IP, three times/week, for week 9 followed by 1 µg IP, three times/week for weeks 10–12 or 10–16). These are representative Mallory's trichrome stain for liver fibrosis (in blue). All control mice (*n*: 8 at 8-weeks; *n*: 8 at 12-weeks; and *n*: 8 at 16-weeks) developed severe liver fibrosis, while mice receiving the RSK-inbitory peptide (*n*: 8 at 12-weeks; and *n*: 8 at 16-weeks) had no fibrosis or only minimal liver fibrosis (*P<0.01*). F. Analysis of hepatic collagen content by the Sirius red collagen–binding assay showed a ∼2.5 to 3-fold increase in C/EBPβ^+/+^ mice treated with CCl_4_ (*n*: 8 for 8 weeks; *n*: 8 for 12 weeks and *n*: 8 for the 16 weeks), compared to animals also receiving the RSK-inbitory peptide (*n*: 6 for 12weeks; *n*: 8 for 16 weeks; *P<0.01*). G. Analysis of hepatic collagen content by the hydroxyproline assay, showed a 2.2-, 3.5- and 5.3-fold increase in C/EBPβ^+/+^ mice treated with CCl_4_, respectively (*n*: 8 for 8 weeks; *n*: 7 for 12 weeks and *n*: 7 for the 16 weeks), compared to animals also receiving the RSK-inbitory peptide (*n*: 6 for 12weeks; *n*: 8 for 16 weeks; *P<0.01*).

Our findings in cell-free, cellular and animal models strongly support the main hypothesis, and suggest a novel, physiological role for unphosphorylated C/EBPβ on caspase 8 activation. Indeed, we found that caspase 8 activation is stimulated by C/EBPβ-Ala217 in HSC from C/EBPβ-Ala217 mice treated with CCl_4 _(Figure 8A). In addition, we determined that the peptide enhanced the activity of recombinant caspase 8 in a cell-free system at picomolar concentrations (Figure 8B). To our knowledge, the C/EBPβ-Ala217 peptides are the first reported compounds to directly enhance caspase 8 activation (G Salvesen, personal communication).

These experiments provided a strong proof-of-principle that RSK activation mediates the signaling required for liver inflammation and liver fibrosis.

### A cell-permeant C/EBPβ-Ala217 peptide stimulates cell death in hepatotoxin-induced activation of HSC

Because in preliminary studies the cell permeant tetrapeptide Ac-KAla217VD-CHO was effective in inducing apoptosis of cultured activated HSC [10], we studied whether this peptide would also be effective in inducing apoptosis of activated HSC in a physiologically relevant animal model of liver injury [10] [16]. To activate HSC, we administered a single dose of CCl_4 _to C/EBPβ^+/+^ (wt) mice, while control mice received the mineral oil vehicle [16]. Six hours later, animals received an intraperitoneal injection of the cell permeant Ac-KAla217VD-CHO peptide [10] (100 µg) or saline vehicle (100 µl). In preliminary studies, we found that 1–100 µg of peptide dose provided adequate bioavailability (M B, unpublished observations). Animals were sacrificed at 24 h. Acute CCl_4_ administration induced both activation and proliferation of HSC (among other hepatic cells), judging by the expression of αSMA and PCNA, as determined by confocal microscopy (Figure 8C and D).

HSC activation and proliferation were blocked by treatment with the Ac-KAla217VD-CHO peptide, but not by treatment with saline (Figure 8C and D). Similarly to our findings of RSK inhibition in cell-free, cultured primary stellate cells and in C/EBPβ-Ala217 transgenic mice, the Ac-KAla217VD-CHO peptide prevented the phosphorylation of C/EBPβ on Thr217 in HSC activated by the liver injury induced by CCl_4_ (Figure 8C). Further, and in agreement to our findings of the increased caspase 8 activation in cell-free, cultured primary stellate cells and in C/EBPβ-A217 transgenic mice, the Ac-KAla217VD-CHO peptide stimulated the apoptotic pathway of C/EBPβ wt HSC as indicated by the presence of active caspase 3 (Figure 8 D).

### A cell-permeant C/EBPβ-Ala217 peptide inhibits progression and stimulates regression of hepatotoxin-induced liver fibrosis

Activation of stellate cells is responsible for the development of liver fibrosis in chronic liver diseases of all causes [3] [4] [8], and remarkably, HSC clearance by apoptosis may allow recovery from liver injury and reversal of liver fibrosis [33] [34].

Given the effective blocking by the RSK-inhibitory peptide of molecular pathways leading to liver fibrosis in an acute CCl_4 _model of liver injury and fibrogenesis, we asked whether these effects would occur in a model of established liver fibrosis due to chronic liver injury, reproducing the disease state of patients with severe liver injury and fibrosis.

Therefore, C/EBPβ^+/+^ mice with severe liver fibrosis, after receiving CCl_4_ for 8 weeks, were treated with the RSK inhibitory peptide for an additional 4 or 8 weeks (5 µg IP, three times/week, for week 9, followed by 1 µg IP, three times/week for weeks 10–12 or 10–16), while continuing to induce liver injury and fibrosis with CCl_4_. Treatment of animals with liver fibrosis with the peptide, while continuing to receive CCl_4_, prevented the progression and induced regression of liver fibrosis compared to control mice treated with CCl_4_. At week-12 or week-16, there was a marked regression of liver fibrosis judging by the trichrome stain (Figure 8E) and Sirius red staining (Figure S5). All control mice (*n*: 22) had severe liver fibrosis, while all mice that received the RSK-inhibitory peptide had minimal or no liver fibrosis. We confirmed these findings by quantitative analysis of liver collagen with the Sirius red binding assay (*P*<0.01) [19] (Figure 8F). Analysis of hepatic collagen content by the hydroxyproline assay (Figure 8G), showed a 2.2-, 3.5-, and 5.3-fold increase in C/EBPβ^+/+^ mice treated with CCl_4_ for 8, 12 and 16 weeks, respectively (*n*: 8 for 8 weeks; *n*: 7 for 12 weeks and *n*: 7 for the 16 weeks), compared to animals treated with the RSK-inhibitory peptide (*n*: 6 for 12 weeks; *n*: 8 for 16 weeks; *P*<0.01).

In agreement with the results observed after CCl_4_ treatment in mice expressing the C/EBPβ-Ala217 transgene, treatment with the RSK-inhibitory peptide of C/EBPβ^+/+^ mice with severe liver fibrosis induced by CCl_4_, while continuing to receive CCl_4,_ reduced the following: i) expression of liver fibrogenic indicators such as collagen α 1 type 1 mRNA (*P*<0.05), α-SMA mRNA (activated HSC) (*P*<0.05), and TGF-β mRNA (fibrogenic cytokine) (*P*<0.01) (Figure 2A, B, and C); and ii) recruitment of CD-68+ inflammatory cells to the liver (Figure 3A).

### Increased expression of active RSK and C/EBPβ-PhosphoThr266 in activated HSC of human liver fibrosis

The RSK phosphoacceptor site in C/EBPβ is identical in mouse and human, it is evolutionarily conserved [5], and necessary for HSC survival upon their activation [10]. The RSK pathway may be critical for HSC activation induced by liver injury, because expression of a catalytically inactive mutant RSK [15], blocked proliferation and survival of cultured HSC upon their activation by collagen type 1 [10].

**Figure 9:**
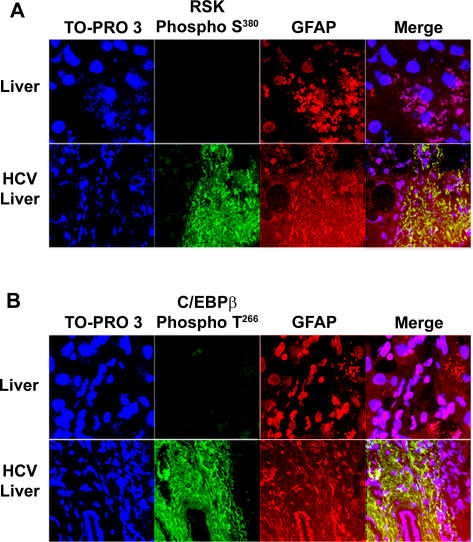
Increased expression of active RSK and C/EBPβ-PhosphoThr217 in activated hepatic stellate cells of human liver fibrosis. Representative confocal microscopy of 4 patients with severe liver fibrosis and 3 control subjects. A. Activated HSC, identified by confocal microscopy for their morphology and fluorescence for glial fibrillary acidic protein (GFAP; red), displayed activated RSK-PhosphoSer380 in livers of patients with severe liver fibrosis (lower panel) but not in the livers of control subjects (upper panel). Colocalization of RSK-PhosphoSer380 and GFAP is shown in yellow (merge). Nuclei are identified with TO-PRO-3 (blue). Only background staining was observed when omitting the first antibody. B. C/EBPβ-PhosphoThr217 (green) was present in activated HSC only in livers of patients with liver fibrosis (lower panel). Colocalization of C/EBPβ-PhosphoThr217 and GFAP (red) is shown in yellow (merge). Nuclei are identified with TO-PRO-3 (blue).

To assess the relevance to human liver fibrosis of the cellular and animal models of liver fibrosis, we analyzed, in preliminary studies, the role of activated RSK and phosphorylated C/EBPβ on Thr266 (identical to mouse Thr217 phosphoacceptor) as possible mechanisms leading to increased liver fibrosis in four patients with chronic hepatitis C viral infection that resulted in severe liver fibrosis (53±17 years) (see Materials and methods). Liver biopsies from hepatitis C patients afflicted with severe liver fibrosis (METAVIR scores of 3 or 4) displayed a high level expression of both active, phosphorylated RSK and phosphorylated human C/EBPβ on Thr266 in activated HSC within the fibrous tissue, compared with samples from three control patients (60±13 years) as identified by confocal scanning microscopy with specific antibodies against RSK-PhosphoSer380, C/EBPβ-PhosphoThr266, and glial fibrillary protein for HSC [10] (Figure 9 A and B).

Thus, our findings in liver biopsies from patients with liver fibrosis are congruent with the hypothesis we developed in cell-free, cellular and animal models that RSK activation and its phosphorylation of C/EBPβ in activated HSC may be important in the development of human liver fibrosis.

## Discussion

Activation of HSC is responsible for the development of liver fibrosis in chronic liver diseases of all causes [3] [4] [8], and remarkably, HSC clearance by apoptosis may allow recovery from liver injury and reversal of liver fibrosis [33] [34].

In this study we show that activation of RSK and phosphorylation of C/EBPβ on Thr217 in activated HSC is critical for the progression of liver fibrosis. We used the classical CCl_4_-induced liver injury and fibrosis model in mice [10] [16], primary mouse and human HSC [10], and cell-free systems to investigate the role of RSK and phosphorylation of C/EBPβ on Thr217 in the activation of HSC and liver fibrosis. It would be important to determine whether RSK and phosphorylation of C/EBPβ are also critical in other animal models that reflect other causes of human liver fibrosis, such as biliary cirrhosis, alcoholic liver disease, immune liver injury and genetic iron overload [3]. Any one of these studies will require as extensive an analysis as that performed with the CCl_4 _model of liver fibrosis.

We found that mice expressing the RSK-inhibitory C/EBPβ-Ala217 transgene were refractory to the induction of HSC activation and proliferation by CCl_4_ treatment. After chronic CCl_4_ administration, C/EBPβ was phosphorylated on Thr217 in HSC of C/EBPβ^+/+^ mice, but not of C/EBPβ-Ala217 mice. C/EBPβ-Ala217 binding to, and blocking, RSK phosphorylation results in decreased phosphorylation of C/EBPβ on Thr217, and presumably, other target survival proteins by activated RSK. Moreover, chronic CCl_4_ treatment induced the apoptotic cascade in HSC in the livers of C/EBPβ-Ala217 mice, but not C/EBPβ^+/+^ mice, as determined by the presence of active caspase 8 and 3.

C/EBPβ-Ala217 was present within the death receptor complex II, with active caspase 8, and was linked to apoptosis of activated HSC, freshly isolated from transgenic mice. After blocking the RSK-C/EBPβ phosphorylation cascade in activated human HSC in culture, with either an ERK1/2 inhibitor or a cell permeant RSK-inhibitory peptide, Ac-KAla217VD-CHO, unphosphorylated C/EBPβ became associated with other members of the death receptor complex II, such as TNFR1, TRAF2, TRADD and RIP [29]. The combined results suggest a functional link between inactive RSK and the active caspase 8 complex II. Further, inactive RSK and active caspase 8 are co-immunoprecipitated with C/EBPβ-Ala217 or unphosphorylated C/EBPβ, suggesting also a physical link. However, identification of a putative RSK/caspase 8 complex would require crystallographic analysis.

In support of our proposed role of unphosphorylated and phosphorylated C/EBPβ-Thr217 on the modulation of HSC survival following their activation by liver injury, expression of the dominant positive phosphorylation mimic C/EBPβ-Glu217 [5] enhances survival of cultured progenitor neuronal cells [35] while C/EBPβ^−/−^ macrophages display defective bacterial killing and tumor cytotoxicity [36]. A corollary of our study is that mice expressing the C/EBPβ-Glu217 transgene would be more susceptible to HSC activation and liver fibrosis induced by liver injury and inflammation. Indeed, this seems to be the case in preliminary studies with these novel transgenic mice (M.B, unpublished observations).

Although the association between C/EBPβ-PhosphoThr217 and inactive procaspase 8 is linked to the inhibition of active caspase 8 [10], the precise molecular mechanisms by which phosphorylated C/EBPβ prevents liver injury-induced HSC apoptosis have not been characterized yet. Phosphorylated C/EBPβ could induce the inhibition of pro-apoptotic proteins, such as p53 [37] or the activation of survival proteins, such as MnSOD [38], or FLIP [29]. Alternatively, granzyme B rather than caspase 8 could be the major target of phosphorylated C/EBPβ-Thr217, as we suggested previously for HSC apoptosis/survival [10].

The hepatotoxin CCl_4_ induced severe liver fibrosis in C/EBPβ^+/+^ mice but not in mice expressing C/EBPβ-Ala217, a non-phosphorylatable RSK-inhibitory transgene as detected by morphological, semi quantitative and quantitative assays. Blocking phosphorylation of C/EBPβ-Thr217 through the inhibition of RSK activity with the C/EBPβ-Ala217 transgene or by C/EBPβ gene knock-out decreases the fibrotic response of the liver to chronic injury. These findings indicate that the RSK phosphoacceptor site in C/EBPβ, which is identical in mouse and human [5], is essential for HSC survival upon their activation in chronic liver injury in mice. Further, the decreased fibrotic response of the liver to the hepatotoxin in C/EBPβ ^−/−^ mice suggests that the critical target of RSK in activated HSC is C/EBPβ-Thr217 rather than other RSK phosphoacceptors in c-Fos, CREB, CBP or other proteins [10]–[14].

Because liver inflammation induces liver injury and liver fibrosis [34] we assessed the degree of liver injury and the inflammatory response to the hepatotoxin. We found decreased liver injury and inflammation in response to CCl_4_ treatment in mice expressing the C/EBPβ-Ala217 transgene. Our data suggest that in addition to increased HSC apoptosis, partial resistance to liver injury and inflammation may contribute to the prevention of liver fibrosis in C/EBPβ-Ala217 mice. A decreased inflammatory response, mediated at least in part by monocytes/macrophages in the livers of C/EBPβ^+/+^ mice, may be responsible for the decreased liver injury in C/EBPβ-Ala217 mice, since RSK inhibition also affected the recruitment of CD-68+ inflammatory cells to the liver. Our results are congruent with the proposed role of macrophages as a major contributor to liver injury and liver fibrosis [20] [22].

Similarly to our findings of RSK inhibition in cell-free, cultured primary stellate cells and in C/EBPβ-Ala217 transgenic mice, the cell permeant Ac-KAla217VD-CHO peptide prevented the phosphorylation of C/EBPβ on Thr217 in HSC activated by the liver injury induced by CCl_4_. Further, and in agreement to our findings of the increased caspase 8 activation in cell-free, cultured primary stellate cells and in C/EBPβ-A217 transgenic mice, the Ac-KAla217VD-CHO peptide stimulated the apoptotic pathway of C/EBPβ^+/+^ HSC as indicated by the presence of active caspase 8 and 3.

C/EBPβ^+/+^ mice with severe liver fibrosis induced by an 8-week CCl_4_ treatment, while continuing on CCl_4_, were treated with the cell permeant RSK-inhibitory peptide for 4 or 8 weeks. The peptide inhibited RSK activation, stimulated apoptosis of HSC and blocked active fibrogenesis, preventing progression and inducing regression of liver fibrosis compared to control mice treated with CCl_4_. Because the resulting activation of HSC and liver fibrosis is similar in any type of chronic liver injury, these results suggest that the inhibition of both RSK and its phosphorylation of C/EBPβ may be effective in preventing/regressing liver fibrosis in animal models that reflect other causes of human liver fibrosis, such as biliary cirrhosis, alcoholic liver disease, immune liver injury, and genetic iron overload [3].

To assess the relevance of the cellular and animal models of liver fibrosis to human liver fibrosis, we analyzed, in preliminary studies, the role of activated RSK and phosphorylated C/EBPβ on Thr266 (identical to mouse Thr217 phosphoacceptor) as possible mechanisms leading to increased liver fibrosis. Liver biopsies from hepatitis C patients afflicted with severe liver fibrosis displayed a high level expression of both active, phosphorylated RSK and phosphorylated human C/EBPβ on Thr266 in activated HSC within the fibrous tissue, compared with samples from control patients. Thus, our findings in liver biopsies from patients with liver fibrosis are congruent with the hypothesis we developed in cell-free, cellular and animal models implicating RSK activation and its phosphorylation of C/EBPβ in activated HSC in the development of human liver fibrosis.

### Clinical Implications

Our data indicate that the RSK-C/EBPβ phosphorylation pathway in HSC is activated in severe human liver fibrosis, and that it is critical for the progression of liver injury to severe liver fibrosis and cirrhosis in an animal model.

This study suggest that blocking RSK activity inhibits fibrogenesis directly by inducing HSC apoptosis, and indirectly, by reducing liver injury and inflammation. There is no available treatment for liver fibrosis. We speculate that these findings may facilitate the development of small molecules potentially useful in the prevention and treatment of liver fibrosis.

Both regression of liver fibrosis as well as lack of progression of liver fibrosis, in spite of continued liver injury, as we clearly documented in our study, are considered important clinical targets for patients with chronic liver disease and liver fibrosis [34]. Finally, blocking the progression of liver fibrosis would decrease development of primary liver cancer in these patients since the majority of hepatocellular carcinomas arise in cirrhotic livers [39].

## Material and Methods

### Construction of C/EBPβ-Ala217 mice

The Animal Protocol was approved by the VA Healthcare Center's Veterinarian Medical Unit. Transgenic mice expressing C/EBPβ-Ala217, a dominant negative, nonphosphorylatable mutation of the C/EBPβ-Thr217 phosphoacceptor, were generated as described previously [10] and back-crossed to the parental wild-type inbreed FVB mice for >4 generations. The mouse C/EBPβ cDNA was amplified by PCR to mutate Thr217. The primers used to mutate Thr217 to Ala217 were S 5′-GCC AAG GCC AAG AAG GCG GTG GAC AAG CTG AGC -3′and AS 5′-GCT CAG CTT GTC CAC CGC CTT CTT GGC CTT GGC -3′. C/EBPβ was removed from pEVRF0 with Apa I and Nhe I and cloned into pHM6 vector (Boehringer-Mannheim, cat. # 1814664) in order to add an RSV promoter upstream to the C/EBPβ start site. C/EBPβ was removed from this new construct with Bsa I and Bsp. The 982 bp insert was cloned into mammalian vector pOP13CAT (Stratagene) from which the CAT portion had been previously removed. DNA for pronuclear injection was purified over a CsCl gradient and digested with Ssp I and EclHK I. The 3.6 Kb fragment was separated on gel electrophoresis on a 0.8% gel with no Ethidium Bromide. The appropriate band was removed and electro eluted from the gel using the Elutrap apparatus from Schleicher & Schuell. The eluted DNA was purified with a Qiagen-20 column, precipitated with isopropanol and dissolved in 7.5 mM TRIS pH 7.4, 0.15 mM EDTA. All solutions were prepared with tissue grade water, endotoxin tested (Gibco). Finally the DNA was dialyzed and injected into fertilized ova at a concentration of 1.8 µg/ml, at the Transgenic Mouse Core Facilities, University of California, San Diego. The presence of the *rsv* gene was used to identify these transgenic mice by PCR, and 3 positive mice resulted. The primer sequences for the RSV PCR were custom designed (RSV.2271 TAGGGTGTGTTTAGGCGAAA sense and RSV.2510 TCTGTTGCCTTCCTAATAAG antisense). The PCR reagents were all from Qiagen. Transgene-bearing founder mice were mated with FVB mice. All founder mice produced viable offspring. This line was bred by mating transgene-positive mice with wild-type FVB and backcrossed to FVB for at least four generations.

### Animal Procedures

In the chronic exposure to the hepatotoxin, C/EBPβ^+/+^, C/EBPβAla217 and C/EBPβ^−/−^ mice [10] [40] (23–27 g) each received intraperitoneal injections of CCl_4_ or mineral oil (50 µl) only once or weekly (for up to 16 weeks). In other chronic experiments, C/EBPβ^+/+^ mice (25 g) each received intraperitoneal injections of CCl_4_ or mineral oil (50 µl) weekly (for up to 16 weeks), but after 8 weeks, animals received either saline (control) or the cell permeant Ac-KAla217VD-CHO (American Peptide) [10] (5 µg IP, three times/week, for week 9, followed by 1 µg IP, three times/week for weeks 10–12 or 10–16) while continuing to induce liver injury and fibrosis with CCl_4_. In the chronic experiments, animals were sacrificed 24 hr after the last CCl_4_ injection

In the acute exposure to the hepatotoxin, animals received the cell permeant Ac-KAla217VD-CHO [10] (100 µg intraperitoneally) at 18 hr and animals were sacrificed at 24 hr [41].

### Cell cultures

Adult C/EBPβ^+/+^ and C/EBPβ-Ala217 transgenic mice were used for the isolation of hepatic stellate cells as described [5] [10]. Stellate cells were prepared, by *in situ* perfusion and single-step density Nycodenz gradient (Accurate Chemical & Scientific Corp., Westbury, New York), as described previously [6]. Stellate cells were identified by their typical autofluorescence at 328nm excitation wavelength, staining of lipid droplets by oil red, and immunohistochemistry with a monoclonal antibody against desmin. Greater than 95% of the isolated cells were stellate cells.

Primary mouse stellate cells freshly isolated from C/EBPβ^+/+^ mice and activated by collagen type 1 matrix [6], were transfected with vectors expressing RSK wild type, a catalytically inactive, dominant negative RSK mutant, or C/EBPβ-Ala217 together with GFP [10]. Transfected stellate cells were selected by sorting for GFP, and cell lysates were immunoprecipitated with C/EBPβ specific antibodies. In other experiments, stellate cells were incubated for 24 hr with an ERK1/2 inhibitor (Calbiochem 328006) (10 µM), or the KAVD peptide (200 µM).

### Microscopy

Fluorescent labels were observed using antibodies against C/EBPβ, RSKPhosphoSer380, α-SMA, PCNA (Santa Cruz Biotechnology, Santa Cruz, California), active caspase 3 (PharMingen, San Diego, California) or C/EBPβ-PhosphoThr217 [10] in a laser confocal microscope [5] [10] [42]. Fluorochromes utilized were Alexa 488 and Alexa 594. At least 100 cells were analyzed per experimental point [41]. We used TO-PRO-3 (Molecular Probes, Eugene, Oregon) to analyze nuclear morphology. The degree of liver fibrosis was determined by using the Mallory's trichrome and the Sirius red Immunohistochemistry [18] [19]. The interobserver agreement was >90%.

### Liver Fibrosis

We determined the degree of liver fibrosis in coded samples. We evaluated liver fibrosis employing the following methods: i) microscopic morphology; ii) semi-quantitative METAVIR grading system [18] ; iii) collagen type 1 immunofluorescence ; iv) quantitative Sirius red collagen-binding assay as described [19] ; v) quantitative hydroxyproline collagen content as described [43] RT-PCR for collagen type 1; vii) RT-PCR for α-smooth muscle actin (present in activated HSC); and viii) RT-PCR for transforming growth factor-β (a pro-fibrotic cytokine) [4] [7] [8].

For measuring hydroxyproline, the liver was excised and a portion was removed and weighed. Hydrolysis of 10 mg tissue/1ml of 6 M HCl was performed at 120°C in a pressure vessel for 4 hr. Samples were dried overnight in a vacuum desiccator, diluted to fall into the assay sensitivity range and internal standards were prepared. The chloramine-T reagent was prepared and 1 ml was added to each sample. The samples were incubated for 20 minutes. One ml of aldehyde-perchloric acid reagent was added to each sample and incubated at 60°C for 15 minutes. Samples were cooled and absorbance was read at 550nm for samples and standards. Concentrations were determined from the standard curve.

### RT-PCR Analysis

We isolated total RNA from control and experimental liver samples using Trizol (Invitrogen). Samples were treated with DNase using Turbo DNA-free (Ambion) and precipitated with chloroform as per manufacturer's protocol. cDNA synthesis was performed using 250 ng of total RNA with the AffinityScript Reverse Transcriptase (Stratagene). Specific sets of primers were utilized for each RT-PCR amplification for collagen α1 type 1, α-SMA and TGF-β, as described by the manufacturer (SuperArray, Frederick, MD).

### Liver Inflammation Genes

The liver expression of 86 inflammation genes was determined by using the RT^2^ Quantitative Real-Time PCR Array as described by the manufacturer (SuperArray, Frederick, MD). Control and experimental liver samples were analyzed together with internal control samples for the RNA purification and amplification steps, as well as for housekeeping genes (β glucuronidase, hypoxanthine guanine phosphoribosyl transferase 1, heat shock protein1β, glyceraldehyde-3-phosphate dehydrogenase, and β-actin), using the BioRad iQ5 real-time PCR detection system (BioRad, Hercules, CA). Isolation of total RNA, treatment with DNase, precipitation with chloroform, and cDNA synthesis was performed using 1 µg of total RNA as described for RT-PCR following the manufacturer's recommendations.

### Cyp-2E1 Expression and Activity

Cyp-2E1 mRNA was measured by RT-PCR in control and experimental liver samples as described above for RT-PCR. Cyp-2E1 protein in liver samples was detected by western blot, following the chemoluminescence protocol (Perkin-Elmer, Shelton, Connecticut) using purified antibodies against Cyp-2E1 (Santa Cruz Biotechnology) as described below.

Cyp-2E1 activity was assessed in a cell-free system, using a recombinant Cyp-2E1 and a specific substrate [44]. Inhibition of Cyp-2E1 activity was achieved using diethyldithiocarbamate (Sigma-Aldrich). We evaluated the effects of the RSK-inhibitory Ac-KAla217VD-CHO peptide (American Peptide) on Cyp-2E1 activity as described per manufacturer (Invitrogen). The standard curve was prepared using dilutions of the fluorescent standard. Master-mix containing the P450 baculosomes reagent, regeneration system, and reaction buffer were added to the designated well of the reaction plate. The plate was incubated for 20 minutes at room temperature to allow the compounds to interact with the Cyp-2E1 in the absence of enzyme turnover. The reaction plate was pre-read at this point to measure any auto-fluorescence. The reaction was initiated with the addition of the reaction mixture containing substrate and NADP. The plate was incubated for 30 minutes, and the fluorescence was measured at 400 and 460 nm. Data analysis was performed as suggested by the manufacturer.

### Immunoprecipitation and Immunoblots

Pre-cleared stellate cell lysates were incubated for 2 h with purified C/EBPβ, RSK, TRADD or caspase 8 antibodies followed by the addition of A/G+ agarose (Santa Cruz Biotechnology) for 12 h. The immunoprecipitation reactions each contained 500 µg of total protein and 2 µg antibody (or purified IgG pre-immune serum as negative control). Immunoprecipitates were washed 3 times in 500 ml cell lysis buffer [45] and resolved by SDS-PAGE, and C/EBPβ, RSK, RSKp380, TNFR-1, TRADD, RIP, TRAF-2, β-actin and caspase 8 detected by western blot [41] [46] [47], following the chemoluminescence protocol (Perkin-Elmer, Shelton, Connecticut) using purified antibodies against C/EBPβ (C-19; aa 258-276), RSK, RSKp380, TRADD, RIP, β-actin (Santa Cruz Biotechnology), procaspase 8 (PharMingen) and C/EBPβ-PhosphoThr217 [10]. Negative samples were performed omitting the first antibody.

### Caspase Activity

Purified synthetic N-acetyl, C-aldehyde KAVD tetrapeptide (American Peptide Company, Sunnyvale, California) were assayed for their ability to enhance the activity of purified human recombinant caspase 8 (catalogue # 201-041-C005) (Alexis Biochemicals). The sequence of caspase 8 includes S217 through D479 cloned into an expression vector containing a 21 amino acid linker at the N-terminus. Thus, the prodomain (first 220 amino acids) is essentially missing. The fragment is cleaved at D385 and the active caspase 8 is essentially identical to that identified in apoptotic cells [48]. Caspase 8 activity was also measured in stellate cell lysates, using recombinant caspase 8 as a standard for activity. Caspase activity was determined by the release of the *p*-nitroanaline colorimetric (Alexis Biochemicals, San Diego, California) substrate for caspase 8 (catalogue # 260-045) within the linear part of the kinetic assay [49] [50]. Active caspase 3 was determined with specific antibodies (PharMingen).

### RSK Activity

RSK activity was measured by the QTL Lightspeed assay (QTL Biosystems; Santa Fe, New Mexico) using purified recombinant RSK-2 (4,333 U/mg) (Upstate, New York), and staurosporine (0.01 nM) as a control inhibitor. RSK activity was measured in the presence or absence of Ac-KThr217VD-CHO (200 µM), Ac-KAla217VD-CHO (200 µM), or C/EBPβ216-253-Ala217 (0.25 nM) peptides.

### Human Livers

We obtained anonymous, de-identified liver samples from 4 patients with chronic hepatitis C viral infection and severe liver fibrosis (Metavir scores of 3 or 4) (53±17 years) and from 3 control subjects without liver disease (60±13 years) (NDRI). The protocol was approved by the University of California, San Diego Human Protection Program.

### Statistical Analysis

Results are expressed as mean (±SD). Either the Student-*t* or the Wilcoxon Mann-Whitney tests were used to evaluate the differences of the means between groups for parametric and non-parametric populations, respectively, with a *P* value of <0.05 as significant.

## Supporting Information

Table S1 C/EBPβ-Ala217 mice have less liver damage after CCl_4_ treatment. Animals received either mineral oil [MO] or CCl_4_. Thirty hours after a single intraperitoneal dose of CCl_4_ C/EBPβ+/+ (n: 9) and C/EBPβ−/− (n: 9) mice had higher serum alanine aminotransferase (ALT) levels than C/EBPβ- Ala217 mice (n: 9) (P<0.005 ). Results are representative of three independent experiments.
(0.02 MB DOC)

Figure S1 Transgenic C/EBPβ-Ala217 and C/EBPβ+/+ mice have similar Cyp-2E1 expression in the liver. Mice were treated with CCl_4_ or control mineral oil for 24 or 72 hr as described in Materials and methods. A. Liver Cyp-2E1 RNA as determined by RT-PCR was comparable at baseline and after CCl_4_ treatment in C/EBPβ +/+ [wt] and C/EBPβ-Ala217 mice (NS). B. Liver Cyp-2E1 protein as determined by western blot was comparable at baseline and after CCl_4_, treatment in C/EBPβ +/+ [wt] and C/EBPβ- Ala217 mice (NS). C. Cyp-2E1 activity as determined in a cell free system using a recombinant Cyp-2E1 protein was comparable in the absence or presence of the RSK-inhibitory Ac-KAVD-CHO peptide (1-1000 ng/ml). Values are expressed as percentage of control Cyp-2E1 activity (open bars) (NS). A specific Cyp-2E1 inhibitor was used as a positive control (closed bar).
(0.66 MB TIF)

Figure S2 The association of C/EBPβ with active caspase 8, TNFR1, TRAF2, TRADD and RIP is induced by inhibiting RSK in HSC. A Reciprocal TRADD immunoprecipitation of experiment described in (7A) confirmed the association of unphosphorylated C/EBPβ with active caspase 8. β - Actin was used as an internal control for the immunoprecipitations. B. Reciprocal TNFR1 immunoprecipitation of experiment described in (Fig. 7B) confirmed the association of unphosphorylated C/EBPβ with TNFR1. β- Actin was used as an internal control for the immunoprecipitations. C. Reciprocal TRAF2 immunoprecipitation of experiment described in (Fig. 7C) confirmed the association of unphosphorylated C/EBPβ with TRAF2. β- Actin was used as an internal control for the immunoprecipitations.
(0.56 MB TIF)

Figure S3 Primary mouse hepatocytes are refractory to the induction of apoptosis by the RSK-inhibitory Ac-KAVD-CHO peptide. Primary mouse hepatocytes were isolated from C/EBP β +/+ [wt] mice as described in Materials and methods. Cells were treated with Ac-KAVD-CHO peptide for 18 hr. Apoptosis was measured by the annexin-V binding assay as described in Materials and methods. The peptide did not induce hepatocyte apoptosis when compared to control hepatocytes (NS).
(0.18 MB TIF)

Figure S4 The RSK-inhibitory peptide blocks liver fibrosis induced by CCl_4_. Mice were from the experiment described in (Fig 8E and F). Representative Sirius red immunohistochemistry for collagen (in red; arrowhead). Marked increase in liver collagen in a cirrhotic pattern was observed in C/EBPβ +/+ treated with CCl_4_ but not in animals receiving the RSK-inbitory peptide.
(0.18 MB TIF)
