# Supplementary material for: A Ribosomal S-6 Kinase–Mediated Signal to C/EBP-β Is Critical for the Development of Liver Fibrosis
Source: PLoS One. 2007 Dec 26;2(12):e1372. doi: 10.1371/journal.pone.0001372 (PMC2137951; doi:10.1371/journal.pone.0001372)
Supplement: Table S1 — C/EBPβ-Ala217 mice have less liver damage after CCl4 treatment. Animals received either mineral oil [MO] or CCl4. Thirty hours after a single intraperitoneal dose of CCl4 C/EBPβ+/+ (n: 9) and C/EBPβ−/− (n: 9) mice had higher serum alanine aminotransferase (ALT) levels than C/EBPβ- Ala217 mice (n: 9) (P<0.005 ). Results are representative of three independent experiments. (0.02 MB DOC) [file pone.0001372.s001.doc]

**Table S 1. C/EBPβ- Ala217 mice have less liver damage after CCl_4_ treatment.**

| **Group** | **Serum ALT (IU/ml)** |
| --- | --- |
| C/EBPβ^+/+^ + M.O. | 9.6 +/- 3 |
| C/EBPβ^-/-^ + M.O. | 8.4 +/- 3 |
| C/EBPβ-Ala217 + M.O. | 7.2 +/- 2 |
| C/EBPβ^+/+^ + CCl_4_ | 11,238 +/- 427 |
| C/EBPβ^-/-^ + CCl_4_ | 10,896 +/- 960 |
| C/EBPβ-Ala217 + CCl_4_ | 2,400 +/- 727 |

Animals received either mineral oil [MO] or CCl_4_. Thirty hours after a single intraperitoneal dose of CCl_4_, C/EBPβ^+/+^ (*n*: 9) and C/EBPβ^-/-^ (*n*: 9) mice had higher serum alanine aminotransferase (ALT) levels than C/EBPβ- Ala217 mice (*n*: 9) (P < 0.005 ). Results are representative of three independent experiments.
