# Supplementary figures and images for: A Ribosomal S-6 Kinase–Mediated Signal to C/EBP-β Is Critical for the Development of Liver Fibrosis
Source: PLoS One. 2007 Dec 26;2(12):e1372. doi: 10.1371/journal.pone.0001372 (PMC2137951; doi:10.1371/journal.pone.0001372)

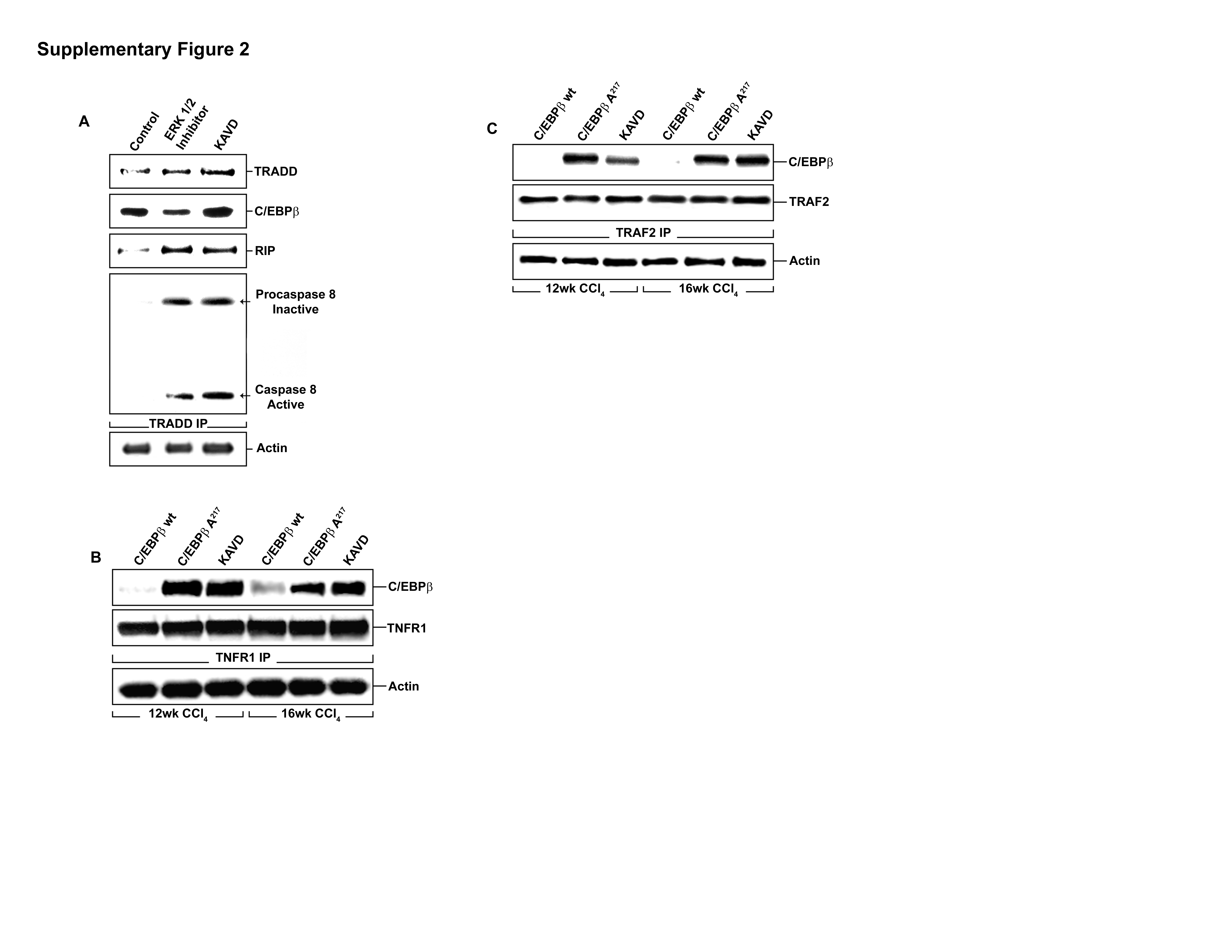

Supplement: Figure S2 — The association of C/EBPβ with active caspase 8, TNFR1, TRAF2, TRADD and RIP is induced by inhibiting RSK in HSC. A Reciprocal TRADD immunoprecipitation of experiment described in (7A) confirmed the association of unphosphorylated C/EBPβ with active caspase 8. β - Actin was used as an internal control for the immunoprecipitations. B. Reciprocal TNFR1 immunoprecipitation of experiment described in (Fig. 7B) confirmed the association of unphosphorylated C/EBPβ with TNFR1. β- Actin was used as an internal control for the immunoprecipitations. C. Reciprocal TRAF2 immunoprecipitation of experiment described in (Fig. 7C) confirmed the association of unphosphorylated C/EBPβ with TRAF2. β- Actin was used as an internal control for the immunoprecipitations. (0.56 MB TIF) [file pone.0001372.s003.tif]

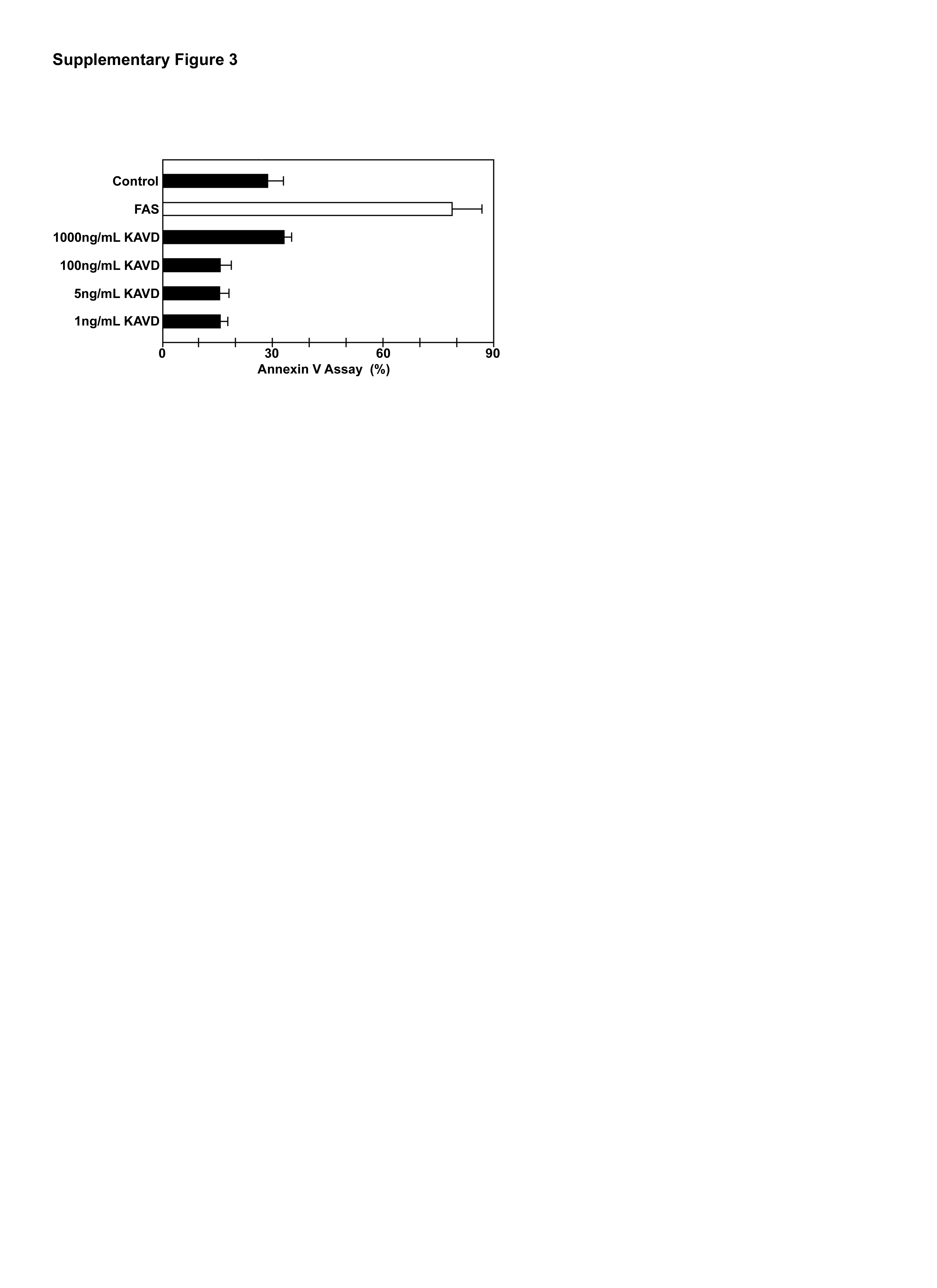

Supplement: Figure S3 — Primary mouse hepatocytes are refractory to the induction of apoptosis by the RSK-inhibitory Ac-KAVD-CHO peptide. Primary mouse hepatocytes were isolated from C/EBP β +/+ [wt] mice as described in Materials and methods. Cells were treated with Ac-KAVD-CHO peptide for 18 hr. Apoptosis was measured by the annexin-V binding assay as described in Materials and methods. The peptide did not induce hepatocyte apoptosis when compared to control hepatocytes (NS). (0.18 MB TIF) [file pone.0001372.s004.tif]
